# Antibiotic Prophylaxis for Surgical Site Infection in General Surgery: Oncological Treatments and HIPEC

**DOI:** 10.3390/antibiotics11010043

**Published:** 2021-12-30

**Authors:** Carlo Vallicelli, Federico Coccolini, Massimo Sartelli, Luca Ansaloni, Simona Bui, Fausto Catena

**Affiliations:** 1 General, Emergency and Trauma Surgery Department, Bufalini Hospital, 47521 Cesena, Italy; fausto.catena@auslromagna.it; 2 General, Emergency and Trauma Surgery Department, Pisa University Hospital, 56124 Pisa, Italy; federico.coccolini@gmail.com; 3 Department of Surgery, Macerata Hospital, 62100 Macerata, Italy; massimosartelli@gmail.com; 4 Department of General and Emergency Surgery, Policlinico San Matteo, 27100 Pavia, Italy; aiace63@gmail.com; 5 Department of Clinical Oncology, Maggiore Hospital, 43126 Parma, Italy; sbui@ao.pr.it

**Keywords:** HIPEC, surgical oncology, antibiotic prophylaxis, surgical site infection

## Abstract

The procedure of cytoreductive surgery (CRS) and hyperthermic intraperitoneal chemotherapy (HIPEC) is a combined surgical and oncological treatment for peritoneal carcinomatosis of various origins. Antibiotic prophylaxis is usually center-related and should be discussed together with the infectious disease specialist, taking into account the advanced oncologic condition of the patient, the complexity of surgery—often requiring multiorgan resections—and the risk of post-HIPEC neutropenia. The incidence of surgical site infection (SSI) after CRS and HIPEC ranges between 11 and 46%. These patients are also at high risk of postoperative abdominal infections and septic complications, and a bacterial translocation during HIPEC has been hypothesized. Many authors have proposed aggressive screening protocols and a high intra and postoperative alert, in order to minimize and promptly identify all possible infectious complications following CRS and HIPEC.

## 1. Antibiotic Prophylaxis in Cytoreductive Surgery and HIPEC: Background and Current Evidence

The procedure of cytoreductive surgery (CRS) and hypertermic intraperitoneal chemotherapy (HIPEC) is a combined surgical and oncological treatment for peritoneal carcinomatosis of various origins. In recent years, a huge amount of literature showing the effectiveness of CRS plus HIPEC has emerged. The treatment is being increasingly used and is recommended in accurately selected patients affected with peritoneal carcinomatosis from colorectal cancer, gastric cancer, ovarian cancer, peritoneal mesothelioma, and pseudomixoma peritonei [1,2,3,4,5,6,7].

CRS is an extensive surgical procedure often requiring multi-organ resection and peritonectomy. Antibiotic prophylaxis is usually center-related and should be discussed together with the infectious disease specialist. The need for colorectal resection and gastric resection should be taken into account, and the choice of prophylactic regimen must ensure coverage of both procedures. Sometimes it is difficult to preoperatively predict the extension of the cytoreduction and the decision to additionally resect colon or stomach can be made intra-operatively. Moreover, patients are exposed to the additional immunosuppression given by the perfusion of the heated chemotherapeutic agent, potentially leading to postoperative neutropenia. Sometimes splenectomy is required together with diaphragmatic peritonectomy due to local invasion, giving the patient an additional risk of immunosuppression [8]. When pelvic carcinomatosis is evident, preoperative ureteral stenting is required, with the potential risk of urinary contamination.

Therefore, several aspects must be evaluated in the choice of antibiotic prophylaxis. Various regimens have been proposed and applied in high-volume institutes, usually involving cephalosporin and metronidazole (especially in case of bowel resection). The incidence of surgical site infection (SSI) after CRS and HIPEC ranges between 11 and 46% [9,10,11]. Full body shower or bathing is recommended at least the night before surgery, whereas hair removal has no indication. Intravenous antibiotics (IVA) are administered within one hour of surgical incision in order to obtain the maximal dose serum level at incision and are repeated according to their pharmacokinetics. The ongoing debates regarding mechanical bowel preparation (MBP) and oral antibiotics (OAB) have never been selectively focused on CRS and HIPEC, but some indications can be assumed from the colorectal literature. Chen et al. reported that MBP together with OA was associated with a lower rate of overall SSI and incisional SSI as compared to MBP alone [12]. Similar results were reported by a review of 38 randomized trials [13]. Moreover, the American College of Surgeons and the Surgical Infection Society support the use of the combination MBP plus OAB [14]. The assumption that OAB is the key element is further supported by the MOBILE (Mechanical and Oral Antibiotic Bowel Preparation Versus no Bowel preparatIon for eLEctive Colectomy) study which found no differences between the two groups in terms of SSI, anastomotic dehiscence, and reoperation rate [15]. According to the ERAS Society guidelines, oral antibiotics together with mechanical bowel preparation can be proposed in patients undergoing CRS and HIPEC in case of probable rectal resection. Preoperative mechanical bowel preparation alone for patients undergoing CRS and HIPEC, including probable colectomy, should not be indicated to reduce the incidence of surgical site infection. Instead, oral antibiotic decontamination with or without preoperative mechanical bowel preparation could be indicated to reduce the incidence of surgical site infection and anastomotic leak [16].

At our institute, we routinely perform antifungal prophylaxis as well, due to the risk of post-HIPEC neutropenia. Moreover, it has to be taken into account that according to ASCO (the American Society of Clinical Oncology) guidelines, antibiotic prophylaxis with fluoroquinolone is recommended for patients who are at high risk of febrile neutropenia or profound, protracted neutropenia. Antifungal prophylaxis with an oral triazole or parenteral echinocandin is also recommended in these patients. Fever in neutropenic patients is defined as a single oral temperature of 38.3 °C or a temperature of 38 °C sustained over a 1-hour period. Neutropenia is defined as an absolute neutrophil count <1000/μL, severe neutropenia as absolute neutrophil count <500/μL, and profound neutropenia as <100/μL. The period of neutropenia is considered protracted if it lasts for seven days or more [17]. The role of *Candida* infections in CRS and HIPEC has been reported in several studies, and is especially relevant and life-threatening in complicated cases requiring reoperation. Capone et al. report a case series of 30 patients undergoing CRS and HIPEC, with five postoperative invasive candidosis recorded. These included four bloodstream infections (three *Candida albicans* and one *C. guillermondii*) and one abdominal candidosis (*C. albicans*). Patients affected by *Candida* infection underwent total peritonectomy and colic resection, had postoperative multiple abscesses—of which two were due to anastomotic leakage and bowel perforation—and underwent reoperation. Four out of the five patients with invasive candidosis died [18].

Several aspects should be taken into account when defining an antibiotic prophylaxis strategy in patients undergoing CRS and HIPEC. Multiple factors, both surgery-related and patient-related, can influence the most appropriate regimen. In this complex setting, there is a shortage of large series specifically addressing this topic and no multicenter randomized trial is available. As a consequence, the decision is often center-related, based on the local expertise of the oncology surgeon and the infectious disease specialist. This situation highlights the need of collaborative research and the spread of data sharing within all the high-volume institutes dedicated to HIPEC surgery.

## 2. Post-HIPEC Infectious Complications: Current Management and Perspectives

Given the role of a proper antibiotic prophylaxis in preventing SSI, oncology surgeons must also be aware of the high risk of postoperative infections requiring antibiotic therapy [19]. Indeed, it is well known that postoperative infections are the most common surgical complication after CRS plus HIPEC. Postoperative morbidity varies between 12 and 52% in the literature. The incidence of infectious complications is particularly elevated and life-threatening. Most patients proposed for CRS and HIPEC have a poor nutritional status, with advanced oncologic disease, and presenting after several cycles of systemic chemotherapy; they show a high risk of endogenous colonization from microorganisms, with a potential role in the following development of infections [20,21].

Some authors have described very aggressive preoperative and postoperative screening protocols in order to reduce the risk of contamination and then the clinical impact of the postoperative infection. Valle et al. reported the results of a prospective protocol including urinalysis and culture, and inguinal, axilla, nasal, and vaginal swabs for preoperative screening. If patients had positive cutaneous sampling, they were submitted to showers with chlorexidine twice a day till negative swabs. Local decontamination of nasal and vaginal positive sampling was performed with tetracycline cream and metronidazole or antifungal vaginal pessaries. If urinary cultures were positive, a directed therapy was started. Postoperatively, cultures of drain tips and fluids, urinalysis and bladder catheter cultures were performed—even if the patient was asymptomatic without fever. In case of symptomatic patients with fever or signs of infections, not only blood cultures from central and peripheral veins were taken, but cultures from bronchial secretions, the thoracic fluid, and CVC tips were also obtained—if present—together with a CT-scan. Empiric antibiotic therapy was promptly started, including carbapenemic and teicoplanine, and was then modified according to the microbiological results. In candidemias, the focus of infection was investigated by means of blood cultures, echocardiogram, and a dilated eye exam—possibly associated to diagnostic imaging. In the authors’ experience, colonic resection, Infection Risk Score, and the duration of surgery are statistically related to postoperative infections. An aggressive protocol of prevention, surveillance, and treatment of postoperative infections led the authors to zero mortality from infection complications in 111 consecutive patients treated with CRS and HIPEC [22].

Interestingly, in case of reoperation after CRS and HIPEC, bacterial cultures from intra-operative samples are often positive—regardless of the reason for surgery (infectious or no infectious complication). Moreover Honore’ et al. have reported that 13% of postoperative peritonitis after CRS and HIPEC happens without an underlying digestive perforation [23]. Therefore, some authors have hypothesized a bacterial translocation during HIPEC as the reason for these postoperative septic complications, assuming that traditional antibiotic prophylaxis may be not enough in this complex procedure. Dazza et al. report a series of 75 consecutive patients undergoing three samples of 20 cc of rinsing liquid from the right and left upper abdominal quadrant and pelvis at the end of HIPEC perfusion performed with the Coliseum technique. Cefotixin was started as the preoperative antibiotic prophylaxis for every patient and stopped as soon as the sample culture appeared negative. In case of a negative aerobic culture result, cefoxitin was switched to metronidazole while waiting for the anaerobic results. Directed therapy was started for patients with positive sample culture results. Thirty (40%) patients had at least one positive intra-operative sample. The cultures were monobacterial in 57% of cases, and pathogens of the digestive flora were found in most cases. Risk factors for positive cultures were colorectal resections and blood loss greater than 1000 cc. Among 26 patients experiencing a postoperative abdominal infectious complication, 13 had isolated complications. A positive intra-operative sample was independently associated with abdominal infectious complications and isolated abdominal infection complications without an underlying digestive or urologic fistula. The authors support the utility of intra-operative bacterial rinsing liquid cultures, especially in patients at high risk for infectious complication due to blood loss, extent of cytoreduction, or colorectal resection [24].

Infections in CRS and HIPEC are a complex chapter, and the relationship between antibiotic prophylaxis and postoperative antibiotic therapy requires strong attention. The HIPEC patient is at high risk of postoperative infections, and a bacterial translocation during the hypertermic perfusion has been hypothesized as the potential cause. Indeed, the rate of postoperative peritonitis without an underlying digestive perforation is impressively high and suggests an additional reflection on the role of standard antibiotic therapy. Further data are required on this topic, but for sure surgeons must maintain a high postoperative after CRS and HIPEC. Aggressive cultures, early CT scan, and a rapid escalation in the antibiotic regimen are all strategies strongly recommended by the most recent literature—especially in high-risk patients [25,26].

Several research perspectives could also be investigated in this topic. For instance, a possible role of Helicobacter Pylori in the disease progression of Pseudomixoma Peritonei has been hypothesized. A treatment regimen of lansoprazole, amoxicillin, and clarithromycin for 14 days—3 weeks prior to CRS/HIPEC—and a second course 2–3 months post-operatively has been proposed, assuming that if bacteria were involved with disease progression, antibiotics could help reduce the bacterial load and positively impact morbidity and mortality. Further studies and additional analyses are currently ongoing [27].

## 3. Conclusions

To conclude, CRS and HIPEC is a complex procedure performed in advanced oncologic patients, and abdominal infectious complications are the most frequently encountered complications in the postoperative course. The role of antibiotic prophylaxis has been strongly debated, and many authors argue that a “simple” pre and intra-operative prophylaxis may be not sufficient in these patients due to the high risk of endogenous colonization from microorganisms with a potential role in the following development of infections. Verwaal et al. reported that infections were the first cause of severe complications and that abdominal sepsis was the first indication for relaparotomy [28]. Capone et al. reported a 37% rate of post-operative infectious complications, with a median of 2.6 infections per patient. Infectious complications were found to be statistically responsible for increased hospital stay lengths and higher mortality rates (36.4 vs. 5%) [18]. Several risk factors for post-operative abdominal infections have been reported—particularly the extent of cytoreduction, colorectal resections, blood loss, and duration of surgery. In these situations, intra-operative samples have been proposed at the end of HIPEC perfusion. Moreover, surgeons must maintain a high alert during the post-operative course. Multiple cultures, abdominal CT scans, and an escalation in antibiotic regimens should be promptly performed as soon as signs and symptoms of infection become evident. A strong collaboration between oncologists, surgeons, and infectious disease specialists is the key to minimizing complications in every high-volume HIPEC institute.
